# Intratumoral and peritumoral post-irradiation changes, but not viable tumor tissue, may respond to bevacizumab in previously irradiated meningiomas

**DOI:** 10.1186/s13014-015-0446-0

**Published:** 2015-07-30

**Authors:** Motomasa Furuse, Naosuke Nonoguchi, Shinji Kawabata, Tomo Miyata, Taichiro Toho, Toshihiko Kuroiwa, Shin-Ichi Miyatake

**Affiliations:** Department of Neurosurgery, Osaka Medical College, 2-7, Daigakumachi, Takatsuki, Osaka 569-8686 Japan

**Keywords:** Anaplastic, Bevacizumab, Meningioma, Radiation necrosis

## Abstract

The efficacy of bevacizumab has not been determined for treatment-refractory meningiomas. We treated meningiomas with low-dose bevacizumab and compared the radiological responses of non-irradiated meningiomas with previously irradiated meningiomas. In addition, we assessed intraparenchymal radiation necrosis following bevacizumab treatment.

Six patients with meningiomas (three anaplastic, one atypical, and two grade I) who were previously treated with multiple sessions of radiotherapy and subsequently developed perilesional edema were treated with bevacizumab. Of six patients, two patients with anaplastic meningiomas developed three tumors following radiotherapy, which were defined as non-irradiated tumors. There were 12 pre-existing extra-axial tumors that were previously irradiated. Some of these tumors demonstrated adjacent intraparenchymal contrast enhancement. These tumors were defined as post-irradiated tumors. Four patients had intraparenchymal radiation necrosis. Low-dose bevacizumab was administered biweekly over 3–6 cycles to all patients.

Four tumors decreased in contrast-enhanced volume, nine tumors were unchanged, and two tumors progressed. Of the three non-irradiated tumors, two tumors increased in volume (126 % and 198 %) and one tumor was stable (−5 %). The median reduction rates determined by contrast volume were −31 % and −71 % in post-irradiated tumors and radiation necrosis, respectively. Non-irradiated tumors had a significantly poorer response to bevacizumab than post-irradiated tumors and radiation necrosis (*p* = 0.0013 and *p* = 0.0005, respectively, Tukey-Kramer test).

Low-dose bevacizumab did not demonstrate efficacy in the treatment of non-irradiated meningiomas. Responses to low-dose bevacizumab could be related to its effect on post-irradiation changes, rather than its effect on biologically active tumor tissue in post-irradiated meningiomas. Radiological responses to low-dose bevacizumab may distinguish biologically active tumors from post-irradiation changes in progressive meningiomas following radiotherapy.

## Background

High-grade meningiomas and benign skull base meningiomas are difficult to treat with surgical resection alone; therefore, these meningiomas are usually treated with surgical resection in combination with radiotherapy. In high-grade meningiomas, the efficacy of standard radiotherapy is limited and high-dose radiotherapy, including stereotactic radiosurgery, particle radiotherapy, and repeated irradiation, is indispensable [1–3]. Unfortunately, these high-dose radiotherapies and multiple sessions of radiotherapy increase the frequency of radiation injury, including radiation necrosis. Chemotherapeutic agents are generally ineffective on meningiomas; however, two recent reports have demonstrated that anaplastic meningioma partially shrank after bevacizumab treatment [4, 5]. However, both tumors in this report had been previously irradiated, and intratumoral or peritumoral post-irradiation changes that caused angiogenesis may be mistaken for tumor tissue and incorrectly inferred as a response to bevacizumab. Some reports, including ours, have demonstrated that bevacizumab, which is an anti-vascular endothelial growth factor (VEGF) antibody, is effective in treating radiation necrosis of the brain [6–10]. VEGF is expressed in regions adjacent to necrotic cores of radiation necrosis, promoting angiogenesis and perilesional edema [11, 12]. It has also been reported that VEGF is expressed in meningiomas [13]. Distinguishing viable and active tumor tissues from post-irradiation changes by magnetic resonance (MR) imaging is challenging. Therefore, the true efficacy of bevacizumab treatment for either viable high-grade or treatment-refractory meningiomas remains unknown.

In the present study, we administered low-dose bevacizumab to patients with meningiomas previously treated with radiotherapy for the treatment of recently developed perilesional edema. Our aim of using bevacizumab treatment was initially to reduce perilesional edema and mass effect. At the commencement of bevacizumab treatment, some patients with anaplastic meninigomas developed new tumors, which had not been irradiated. We retrospectively evaluated the response of non-irradiated anaplastic meningiomas to bevacizumab. We also compared the response to bevacizumab between non-irradiated tumors and previously irradiated tumors, which may include intratumoral or peritumoral post-irradiation changes.

## Methods

Six patients with meningiomas (three anaplastic, one atypical, and two grade I) were treated with bevacizumab between August 2012 and January 2014. All patients had undergone surgical resection and multiple sessions of radiotherapy, and recently developed massive perilesional edema. There were three non-irradiated tumors that had developed after radiotherapy in two patients with anaplastic meningiomas. These tumors were defined as “non-irradiated tumors.” All patients had tumors that had been previously treated with radiotherapy. Some pre-existing extra-axial tumors were heterogeneously enhanced by contrast media and accompanied by adjacent parenchymal contrast-enhancement. These lesions likely represent both viable tumor tissue and post-irradiation changes and were defined as “post-irradiated tumors.” There were 12 pre-existing extra-axial post-irradiated tumors in six patients. Four patients had intraparenchymal contrast-enhanced lesions that were not contiguous with extra-axial tumors. These lesions were defined as “radiation necrosis.”

The initial aim of bevacizumab treatment was to reduce perilesional edema. Bevacizumab was intravenously administered at a dose of 5 mg/kg bi-weekly over 3–6 cycles. The time between the last radiotherapy session and the commencement of bevacizumab treatment ranged from 2.5 to 17 months (median 6 months). The response to bevacizumab was evaluated using volumetric analysis. The volume of a measurable extra-axial enhanced tumor or a measurable intraparenchymal enhanced radiation necrosis was calculated from the sum of the enhanced area on each slice of gadolinium-enhanced T1-weighted MRI multiplied by the slice thickness. The volume of perilesional edema identified as hyperintense lesions on fluid attenuated inversion recovery (FLAIR) imaging was calculated in the same manner. The reduction rate due to bevacizumab was calculated by dividing the pre-treatment volume subtracted from the post-treatment volume by the pretreatment volume. “Response” was defined as a 50 % reduction (−50 %). “Progression” was defined as a 25 % increase (25 %). Lesions that were categorized as neither “response” nor “progression” (−49–24 %) were defined as “stable.” The study protocol was approved by the Osaka Medical College Ethics Committee.

## Results

Study patient demographics and tumor characteristics are shown in Table 1. Bevacizumab was administered for 6 cycles in two patients, 4 cycles in one patient, and 3 cycles in three patients. There were no apparent adverse events during bevacizumab treatment.

**Table 1:** Demographics of patients and tumors

Case	Age (years old)	M/F	Tumor	Bev (cycles)	Lesion	Location	Radiotherapy	Bev from RT (month)	Reduction Rate		Outcome	
									FLAIR ( %)	Gd ( %)	Radiological	Clinical
Case 1	70	F	anaplastic	4	Tumor 1 (non-irradiated)	R frontal (cranial)		N/A	−71	198	Progression	Improved L hemiparesis
					Tumor 2 (non-irradiated)	R frontal (caudal)		N/A		126	Progression	
					Tumor 3	R frontal	SRT 32Gy	8		−89	Response	
					Tumor 4	R frontotemporal	SRS 23Gy/SRS 20Gy/ SRS 22Gy/ SRT 33Gy	3		7	Stable	
Case 2	52	M	anaplastic	3	Tumor 5	occipital	SRS 14Gy/ SRS 14Gy/ SRT 25.5Gy/BNCT	13	−47	−10	Stable	Unchanged dizziness and visual disturbance
					Tumor 6	L temporal	SRT 27Gy	20		−79	Response	
					Tumor 7 (non-irradiated)	R temporal		N/A		−5	Stable	
					Tumor 8	L tentorial edge	SRS 20Gy	2.5		−55	Response	
Case 3	76	F	atypical	6	Tumor 9	parasagittal	SRT 24Gy/ BNCT	8	−29	0	Stable	Unchanged L hemiparesis
Case 4	52	M	grade I	3	Tumor 10	petroclival	GKR 14Gy/RT 30Gy/GKR 14Gy	5.5	−33	−14	Stable	Improved R hemiparesis and L ataxia
Case 5	69	F	grade I	3	Tumor 11	parasagittal	SRS/ SRS	13	−90	−47	Stable	Improved gait disturbance
Case 6	46	F	anaplastic	6	Tumor 12	frontal base	EBRT 60Gy/ BNCT/BNCT	4	−45	−48	Stable	Unchanged cognitive dysfunction
					Tumor 13	orbit	BNCT/BNCT	4		−65	Respose	
					Tumor 14	infratemporal	BNCT/BNCT	4		12	Stable	
					Tumor 15	frontal convexity	EBRT 60Gy/ BNCT/BNCT	4		19	Stable	

**Fig. 1:**
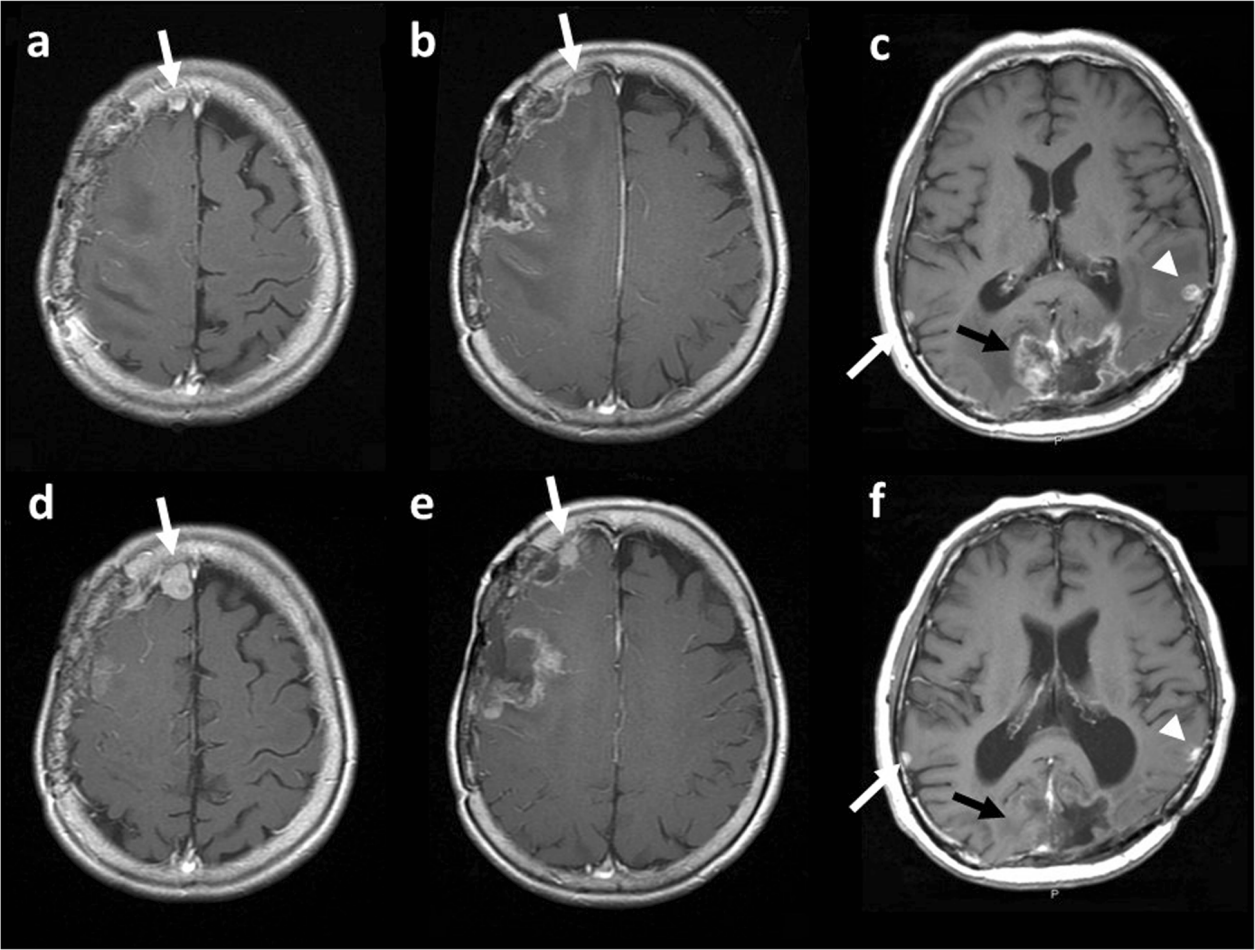
MR images before and after bevacizumab in Case 1 (before, a, b; after, d, e) and Case 2 (before, c; after, f). There were three non-irradiated tumors (**a**, white arrow: tumor 1; **b**, white arrow: tumor 2; **c**, white arrow: tumor 7). After bevacizumab, both Tumors 1 and 2 showed progression (**d**, **e**: arrows). Tumor 7 did not changed after bevacizumab treatment (**f**: arrow). On the other hand, a post-irradiated tumor decreased after bevacizumab. Tumor 6 decreased to −79 % (c, f: arrowheads). Intraparenchymal radiation necrosis was disappeared (c, f: black arrow)

**Fig. 2:**
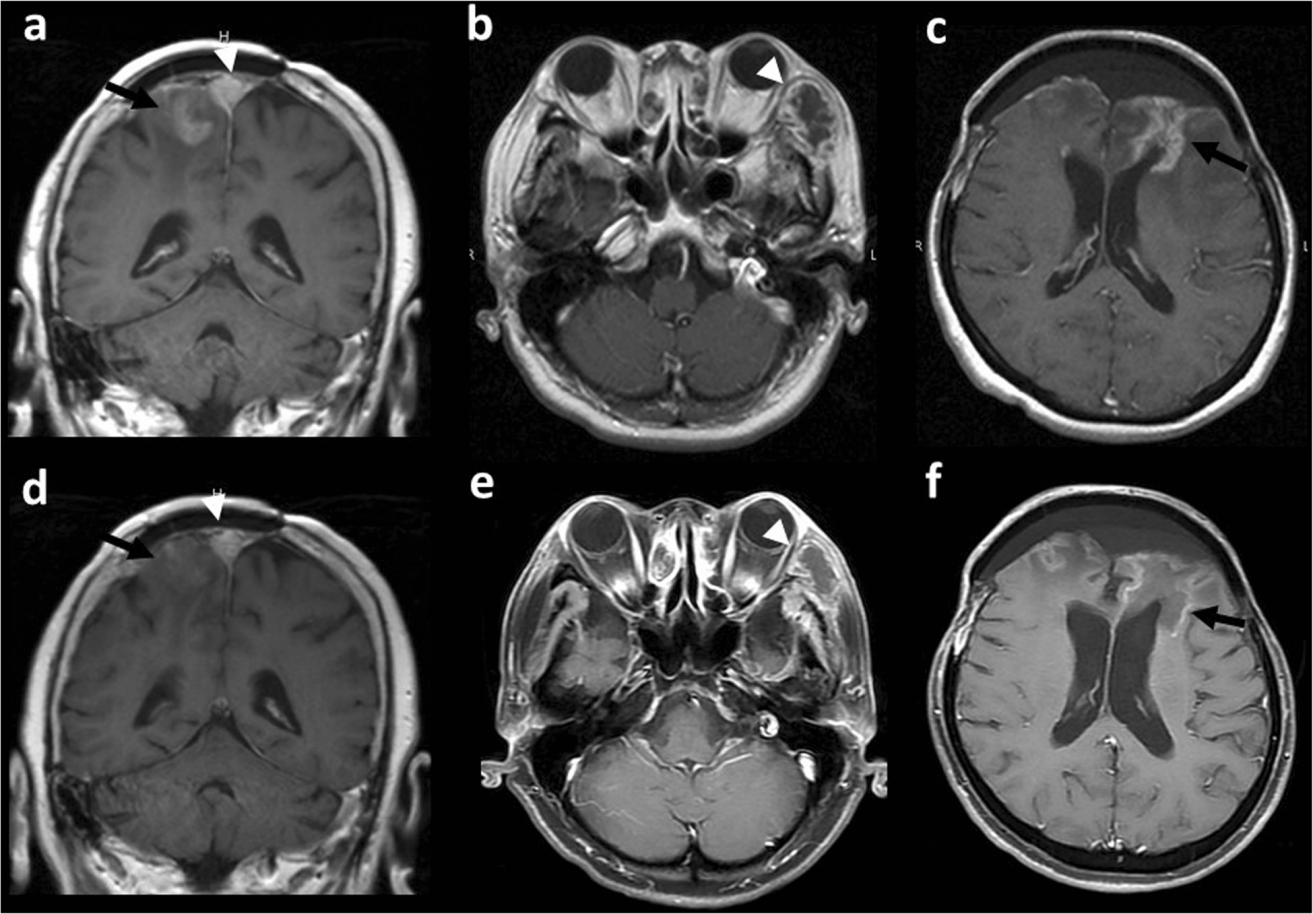
MR images before and after bevacizumab in Case 3 (before, **a**; after, **d**) and Case 6 (before, **b**, **c**; after, **e**, **f**). Intraparenchymal radiation necrosis decreased to −65 % and −51 % after bevacizumab in Cases 3 and 6, respectively (black arrows). Regarding post-irradiated tumors, Tumor 9 (Case 3) (a, d, arrowhead) did not change (0 %) and Tumor 13 (Case 6) (b, e, arrowhead) showed 65 % decrease after bevacizumab

**Fig. 3:**
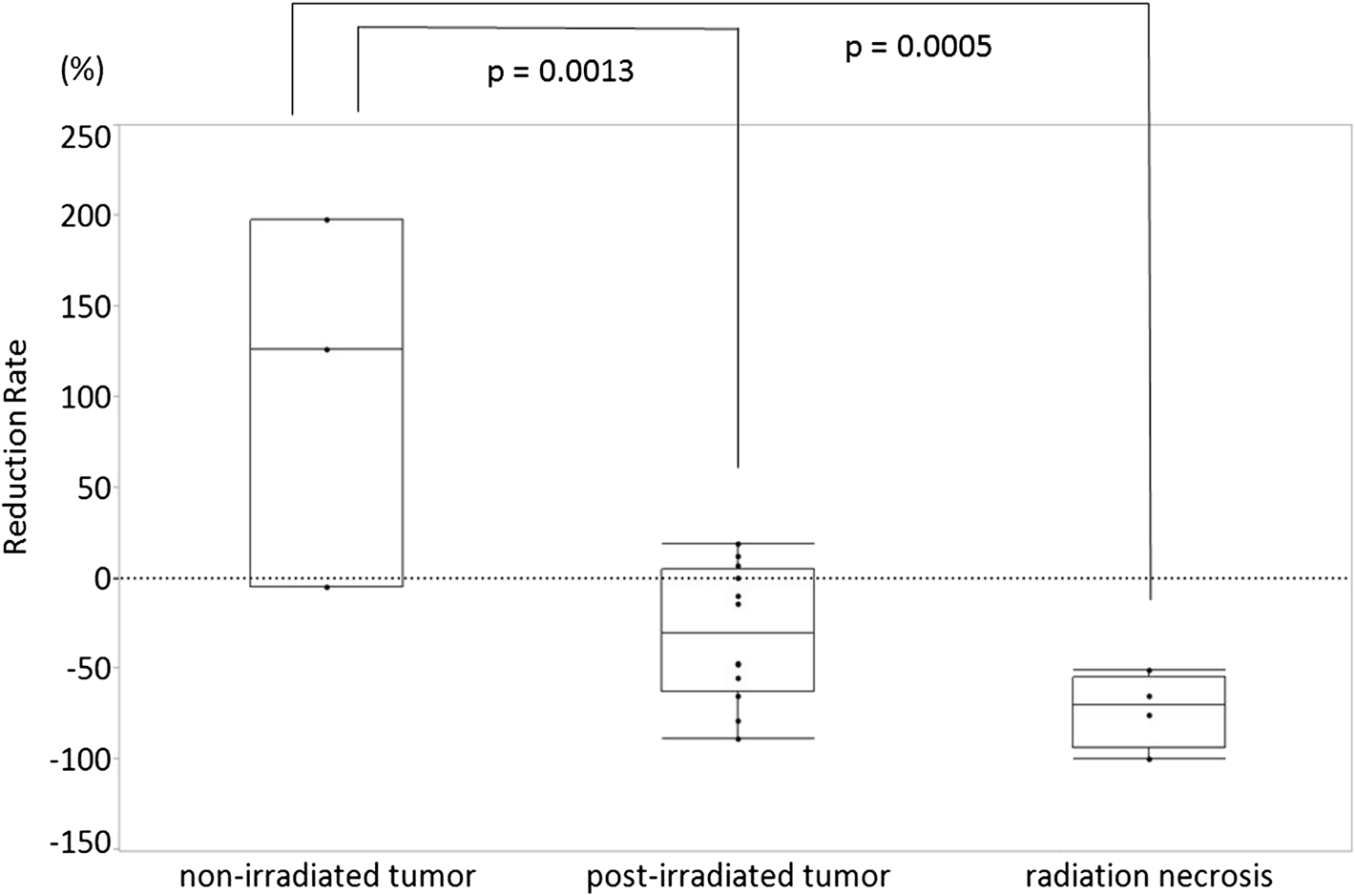
Graph demonstrating the reduction rate of non-irradiated meningiomas, post-irradiated meningiomas, and intraparenchymal radiation necrosis. Non-irradiated tumors responded significantly poorer than post-irradiated tumors and radiation necrosis (*p* = 0.0013, *p* = 0.0005, respectively, Tukey-Kramer test)

In all patients, perilesional edema improved following bevacizumab treatment. Perilesional edema reduced by between −90 % and −29 % compared to pretreatment edema (median −46 %). The median change in contrast enhanced tumor volume of 15 meningiomas in response to bevacizumab was −10 % compared to pretreatment tumor volumes (range −89–198 %). Four tumors demonstrated a response according to tumor volume, nine tumors were stable, and two tumors progressed. Of three non-irradiated tumors, two tumors in Case 1 progressed (126 % and 198 %, respectively) (Fig. 1a, b, d, e) and one tumor in Case 2 was stable (−5 %) (Fig. 1c, f). On the other hand, all post-irradiated tumors were controlled by bevacizumab (Fig. 1c, f, Fig. 2a, b, d, e). The median reduction rate of post-irradiated tumors was −31 % (−89–19 %). With regard to radiation necrosis, contrast-enhanced intraparenchymal lesions were decreased in all patients (Fig. 1c, f, Fig. 2a, c, d, f). The median reduction rate of radiation necrosis was −71 % (−100–−51 %) (Table 2). There was a significant difference of the reduction rate between non-irradiated tumors, post-irradiated tumors, and radiation necrosis (*p* = 0.0005, ANOVA) (Fig. 3). Non-irradiated tumors responded significantly poorer than post-irradiated tumors and radiation necrosis (*p* = 0.0013 and *p* = 0.0005, respectively, Tukey-Kramer test). However, there was not a significant difference between post-irradiated tumors and radiation necrosis (*p* = 0.3155, Tukey-Kramer test). Clinically, the symptoms of three patients improved after bevacizumab, whereas those of the other three patients did not change.

**Table 2:** Summary of intraparenchymal radiation necrosis

Patients	Age (years old)	Gender	Tumor	Bevacizumab (cycles)	Lesion	Location	Radiotherapy	Bev from RT (month)	Response rate ( %)	Radiological assessment
Case 2	52	M	anaplastic	3	Necrosis 1	occipital	SRS 14Gy/ SRS 14Gy/ SRT 25.5Gy/BNCT	13	−100	Response
Case 3	76	F	atypical	6	Necrosis 2	parasagittal	SRT 24Gy/ BNCT	8	−65	Response
Case 5	69	F	grade I	3	Necrosis 3	parasagittal	SRS/ SRS	13	−76	Response
Case 6	46	F	anaplastic	6	Necrosis 4	frontal	EBRT 60Gy/ BNCT/BNCT	4	−51	Response

## Discussion

High-grade meningiomas and deep-seated benign meningiomas are difficult to manage. Any chemotherapeutic agent had not been shown to be effective on meningiomas [14–16]. Recently, there were some reports that bevacizumab was used for meningiomas (Table 3) [4, 5, 17–20]. Lou et al. reported 14 cases of meningiomas treated with bevacizumab [5]. Ten of 14 patients had undergone prior radiotherapy, and bevacizumab was used as monotherapy in four patients and in combination with other chemotherapeutic agents in 10 patients. Treatment efficacy was evaluated using the Response Assessment in Neuro-Oncology [21]. There was no complete response (CR), but a partial response (PR) in one patient, stable disease in 11 patients, and progressive disease in two patients were observed. The authors noted that there was a general trend toward increased progression-free survival in patients who had received stereotactic radiotherapy. In another case series, Nayak L et al. reported that grade II and III meningiomas were treated with bevacizumab [18]. Neither CR nor PR was obtained, and the best response was stable disease. There are two more case reports demonstrating that bevacizumab was used for malignant meningiomas [4, 20]. Both tumors had already irradiated before bevacizumab. The tumor partially responded to bevacizumab in one case and multiple tumors were stable or slowly growing after bevacizumab in the other case. There was no report that a non-irradiated anaplastic meningioma responded to bevacizumab. Nunes et al. treated neurofibromatosis type 2-related meningiomas with bevacizumab [19]. Fourteen patients had progressive vestibular schwannomas and coexisting meningiomas and one patient had progressive meningioma. They retrospectively analyzed the response to bevacizumab and found that tumor volume was reduced by 20 % or more in 29 % of meningiomas. [4, 17, 20]. Goutagny et al. also reported on the response of a coexisting convexity meningioma to bevacizumab in a patient with neurofibromatosis type 2 (the target of bevacizumab treatment was a vestibular schwannoma) [17]. The tumor size was reduced by 22 % after bevacizumab. VEGF expression tended to increase with tumor grade; however, the difference did not reach statistical significance [22, 23]. Conversely, the degree of VEGF expression has been reported to have a positive correlation with the extent of peritumoral edema [22, 24].

**Table 3:** Review of literatures reporting bevacizumab treatment for meningiomas

Authors	Tumor	Prior Tx	Bevacizumab	Treatment duration	Radiological assessment	Response	PFS/OS	Toxicity
Putchner MJA, et al. 2010 [4]	1 Anaplstic	60Gy RT	10 mg/kg every 2 weeks	6 months	N/A	1 PR	N/A	hypertension
Goutagny S, et al. 2011 [17]	7 tumors in NF2 pts	none	5 mg/kg Every 2 weeks	15 months	volume	1 tumor: 22 % decrease	N/A	No mention
						6 tumors N/C		
Lou E, et al. 2012 [5]	5 GI	RT: 12/14	10 mg/kg very 2 weeks	0.5–29.5 months	RANO	0 CR	mPFS: 17.9 months	Tumoral hemorrhage
	5 GII	CT: 11/14				1 PR	PFS-6: 85.7 %	G4 intestinal perforation
	3 GIII					11 SD		G5 pneumonia/sepsis
	1 unk					2 PD		
Nayak L, et al. 2012 [18]	6 GII	RT: 15/15	10 mg/kg every 2 weeks	9 doses (1–19 doses)	RANO	0 CR	mPFS: 26 weeks	G1 Intratumoral hemorrhage
	9 GIII	CT: 7/15				0 PR	mOS: 15 months	G2 fatigue
						15 SD		
Nunes FP, et al. 2013 [19]	48 tumors in 15 NF2 pts		5 mg/kg every 2 weeks		Response: 20 % reduction	Tumor: 29 % response	Per-tumor	4 G3 events
					Progression: 20 % increase	Patients	PFS-6: 85 %	2 G4 events (wound healing problems)
						7 % response	Per-patient	
							PFS-6: 93 %	
Boström JP, et al. 2014 [20]	1 GIII	60Gy RT	5 mg/kg every 2 weeks	2 months	N/A	Stable and growing	N/A	No mention
		35Gy SRT						

All of anaplastic meningiomas treated with bevacizumab in these reports were previously treated with radiotherapy. Our study had three non-irradiated anaplastic meningiomas and these tumors were not responded. Post-irradiated tumors responded to bevacizumab significantly better than non-irradiated tumors. Likewise, intraparenchymal radiation necrosis also significantly responded better than non-irradiated tumors. Therefore, we presume that enhancement by contrast, which is attenuated or disappears after bevacizumab treatment, may be an intratumoral or peritumoral post-irradiation change. Our previous studies have shown that low-dose bevacizumab is also effective for radiation necrosis [9, 10]. Low-dose bevacizumab is enough to reduce angiogenesis caused by post-irradiation changes, including radiation necrosis. In the present study, however, low-dose bevacizumab was not effective for treating non-irradiated anaplastic meningiomas. Post-irradiated tumors responded in various degrees to bevacizumab. The reason was that viable tumor tissue and post-irradiation changes could be coexistent in tumors previously treated with radiotherapy and a section of post-irradiation change may be influenced by reduction rate to bevacizumab. It is difficult to distinguish between these two pathologies in post-irradiated tumors using conventional MR sequences. However, the diagnostic ability of MRI has recently progressed. On diffusion-weighted MRI, the apparent diffusion coefficient reflecting cellular density has been shown to be useful in differentiating post-irradiation effects from tumor recurrence or progression in high-grade gliomas [25]. Dynamic contrast-enhanced MR perfusion study has also shown its utility for distinguishing these two pathologies in gliomas [26]. In a recent study, multiparametric clustering MR imaging data demonstrated greater diagnostic accuracy in differentiating tumor progression from post-treatment change than did a single parameter method such as ADC or each perfusion parameter [27]. In diagnostic nuclear medicine, recurrent and progressive meningiomas show a high uptake of ^18^F-FDG, ^11^C-methionine, and ^18^F-boronophenylalanine on PET [28–30], and PET may differentiate between viable tumor tissues and post-irradiation changes in malignant meningiomas after radiation therapy. However, PET is not as widely available as MRI.

In our series, all patients showed progression of perilesional edema before bevacizumab treatment. In this condition, it is sometimes difficult to judge whether the main cause of perilesional edema is either tumor progression or post-irradiation changes. It could be to be desired that low-dose bevacizumab is firstly used to improve patients status when the lesion is not conclusively diagnosed as either a tumor progression or post-irradiation changes. In our study, half of the patient’s neurological symptoms were ameliorated, reducing perilesional edema. Low-dose bevacizumab is expected to provide clinical benefits in patients with symptomatic perilesional edema irrespective of the pathology of the enhanced lesion. Low-dose bevacizumab could reduce contrast enhancement if a tumor includes post-irradiation changes. Residual enhanced lesion after bevacizumab may be mostly a viable tumor tissue. If bevacizumab is not effective and a large enhanced lesion still exists, then additional radiotherapy or surgical resection could be a treatment option to control disease. Preceding bevacizumab would avoid unnecessary additional radiotherapy for post-irradiated tumor.

## Conclusions

Three non-irradiated anaplastic meningiomas did not respond to low-dose bevacizumab in our study. Some post-irradiated meningiomas responded to bevacizumab. Post-irradiated meningiomas had heterogeneous pathology; residual or recurrent active tumors coexisted with post-irradiation changes. Intratumoral and peritumoral post-irradiation changes, but not viable tumor tissue, may respond to low-dose bevacizumab in previously irradiated meningiomas. MR imaging after low-dose bevacizumab treatment could be used to differentiate active tumors from post-irradiation changes on the basis of the response of contrast enhanced lesions in post-irradiated meningiomas.

### Consent

Written informed consent was obtained from all patients for the publication of this report and any accompanying images.
